# Crystal structure of 4,4′-bis­[3-(piperidin-1-yl)prop-1-yn-1-yl]-1,1′-biphen­yl

**DOI:** 10.1107/S2056989017007277

**Published:** 2017-05-19

**Authors:** Anqi Walbaum, E. Kim Fifer, Sean Parkin, Peter A. Crooks

**Affiliations:** a Dept. of Pharm. Sciences, College of Pharmacy, University of Arkansas for Medical Sciences, Little Rock, AR 72205, USA; b Dept. of Chemistry, University of Kentucky, Lexington KY 40506, USA

**Keywords:** crystal structure, biphenyl system, piperidine ring, bis-tertiary ammonium salt

## Abstract

The title compound, C_28_H_32_N_2_, (I), is one of a second generation of compounds designed and synthesized based on a very potent and selective α9α10 nicotinic acetyl­choline receptor antagonist ZZ161C {1,1′-[[1,1′-biphen­yl]-4,4′-diylbis(prop-2-yne-3,1-di­yl)]bis­(3,4-di­methyl­pyridin-1-ium) bromide}, which has shown analgesic effects in a chemotherapy-induced neuropathy animal model. Compound (I) was synthesized by the reaction of 4,4′-bis­(3-bromo­prop-1-yn-1-yl)-1,1′-biphenyl with piperidine at room temperature in aceto­nitrile. The single-crystal used for X-ray analysis was obtained by dissolving (I) in a mixture of di­chloro­methane and methanol, followed by slow evaporation of the solvent. In the crystal of (I), the biphenyl moiety has a twisted conformation, with a dihedral angle of 25.93 (4)° between the benzene rings. Both piperidine head groups in (I) are in the chair conformation and are oriented so that the N-atom lone pairs of each piperidine group point away from the central biphenyl moiety.

## Abstract 2

The synthesis and structure of the title piperidine derivative is reported. It is one of a second generation of compounds designed and synthesized based on a very potent and selective α9α10 nicotinic acetyl­choline receptor antagonist ZZ161C, which has shown analgesic effects in a chemotherapy-induced neuropathy animal model.

## Chemical context   

The α9α10 nicotinic acetyl­choline receptor is a novel therapeutic target with potential significance for pain management. Previous studies have shown that antagonism of the α9α10 nAChR by the non-peptide small mol­ecule, ZZ161C {10-[(1,1′-biphen­yl)-4,4′-diyl *bis*(prop-2-yne-3,1-di­yl)]*bis*(3,4-di­methyl­pyridin-1-ium) bromide} produced analgesia in the vincristine-induced neuropathic pain model in rats (Zheng *et al.*, 2011 ▸; Wala *et al.*, 2012 ▸). In order to improve the drug-like and pharmacokinetic properties of ZZ161C, the title compound (I) was designed and synthesized. Compound (I) is a biphenyl system with ethynyl appendages at the 4 and 4′ positions, as in ZZ161C, but the terminal aza-aromatic rings have been replaced by piperidine moieties. Single-crystal X-ray analysis of compound (I) was used to determine the structural conformation of the compound.
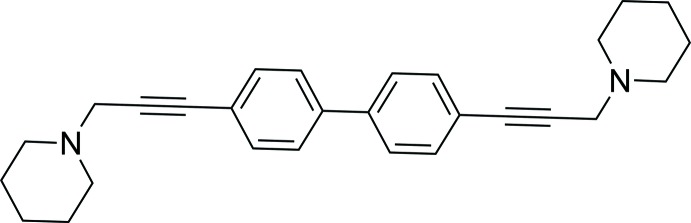


## Structural commentary   

**Figure 1:**
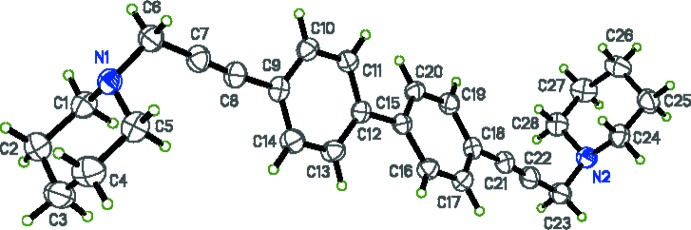
The mol­ecular structure of (I), with ellipsoids drawn at the 50% probability level.

The title compound (I) is shown in Fig. 1 ▸. X-ray crystallographic study was conducted in order to determine the geometry of the biphenyl system as well as to obtain detailed information about the conformation of the terminal piperidine groups. In compound (I), the biphenyl rings (C9-C14) and (C15-C20) are non-coplanar, with a dihedral angle of 25.93 (4)° between them. The torsion angles of the ethynyl groups between the planes of the phenyl rings and the piperidine ring N atoms are 167.49 (9) and 34.01 (12)° (defined by atoms N1/C6/C9/C10, N2/C23/C18/C19, respectively). The lone pair on each N atom is oriented away from the biphenyl core of the mol­ecule.

## Supra­molecular features   

Aside from weak van der Waals inter­actions, there are no noteworthy inter­molecular contacts in (I). The molecules pack into layers in the *ab* plane bounded top and bottom by piperidine groups, which in turn stack along *c*.

## Database survey   

A search of the November 2014 release of the Cambridge Structure Database (Groom *et al.*, 2016 ▸), with updates through May 2015, using the program *Mogul* (Bruno *et al.*, 2004 ▸) for 4,4′-substituted biphenyl fragments was conducted. The search was restricted to purely organic, solvent-free structures with *R* <5% and Cl as the heaviest element. There were over 1000 hits, which produced a bimodal distribution of biphenyl torsion angles with a tight peak at 0° and a broader peak centred at 30°. Therefore the biphenyl torsion angle in (I) is not unusual.

## Synthesis and crystallization   

**Synthetic procedure:** The inter­mediate 4,4′-bis­(3-bromo­prop-1-yn-1-yl)-1,1′-biphenyl (Wan *et al.*, 2015 ▸) was obtained utilizing a previously reported procedure; compound (I) was synthesized by reacting piperidine with this inter­mediate.

To a suspension of 4,4′-*bis*(3-bromo­prop-1-yn-1-yl)-1,1′-biphenyl (100.0 mg, 0.26 mmol) in aceto­nitrile (7 mL), piperidine (66.4 mg, 0.78 mmol) was added at room temperature and the mixture was stirred continuously for 2 h, resulting in the formation of compound (I). Aceto­nitrile was removed under vacuum and the mixture was partitioned between water (50 mL) and di­chloro­methane (50 mL). The di­chloro­methane layer was collected and dried over anhydrous sodium sulfate. Sodium sulfate was removed by filtration, and the filtrate containing crude (I) was concentrated and purified by column chromatography (di­chloro­methane/methanol) to afford pure compound (I) in 80% yield.

**Crystallization:** Light-yellow crystals of compound (I) suitable for X-ray analysis were grown in a mixture of di­chloro­methane and methanol (2:1) by slow evaporation of the solvent at room temperature over a period of 24 h.

^1^H-NMR (400 Mz, CDCl_3_): δ 7.51 (*q*, 8H), 3.53 (*s*, 4H), 2.62 (*s*, 8H), 1.65–1.71 (*m*, 8H), 1.47 (*s*, 4H) ppm.

^1^C-NMR (100 Mz, CDCl_3_): δ 140.07, 132.35, 126.91, 122.54, 85.25, 53.56, 48.62, 25.93, 23.93 ppm.

## Refinement details   

Crystal data, data collection and structure refinement details are summarized in Table 1 ▸. H atoms were found in difference-Fourier maps, but subsequently included in the refinement using riding models, with constrained distances set to 0.94 Å (C*sp* ^2^—H) and 0.98 Å (*R*_2_—CH_2_). *U*_iso_(H) values were set to 1.2*U*_eq_ of the attached carbon atom.

## Supplementary Material

Crystal structure: contains datablock(s) global, I. DOI: 10.1107/S2056989017007277/sj5530sup1.cif

Structure factors: contains datablock(s) I. DOI: 10.1107/S2056989017007277/sj5530Isup2.hkl

Supporting information file. DOI: 10.1107/S2056989017007277/sj5530Isup3.cml

CCDC reference: 1550512

Additional supporting information:  crystallographic information; 3D view; checkCIF report

## Figures and Tables

**Table 1:** Experimental details

Crystal data	
Chemical formula	C_28_H_32_N_2_
*M*_r_	396.55
Crystal system, space group	Monoclinic, *C*2/*c*
Temperature (K)	210
*a*, *b*, *c* (Å)	40.2728 (8), 6.9679 (1), 16.0119 (3)
β (°)	92.588 (1)
*V* (Å^3^)	4488.63 (14)
*Z*	8
Radiation type	Cu *K*α
μ (mm^−1^)	0.51
Crystal size (mm)	0.25 × 0.24 × 0.05

Data collection	
Diffractometer	Bruker X8 Proteum diffractometer
Absorption correction	Multi-scan (*SADABS*; Krause *et al.*, 2015 ▸)
*T*_min_, *T*_max_	0.822, 0.942
No. of measured, independent and observed [*I* > 2σ(*I*)] reflections	28464, 4089, 3656
*R*_int_	0.040
(sin θ/λ)_max_ (Å^−1^)	0.603

Refinement	
*R*[*F* ^2^ > 2σ(*F* ^2^)], *wR*(*F* ^2^), *S*	0.038, 0.120, 1.08
No. of reflections	4089
No. of parameters	272
H-atom treatment	H-atom parameters constrained
Δρ_max_, Δρ_min_ (e Å^−3^)	0.15, −0.14

Computer programs: *APEX2* and *SAINT* (Bruker, 2006 ▸), *SHELXS* (Sheldrick, 2008 ▸), *SHELXL2014* (Sheldrick, 2015 ▸), *SHELXTL* and *XP* in *SHELXTL* (Sheldrick, 2008 ▸) and *CIFFIX* (Parkin, 2013 ▸).

## supplementary crystallographic information

### Crystal data

**Table:**

C_28_H_32_N_2_	*F*(000) = 1712
*M**_r_* = 396.55	*D*_x_ = 1.174 Mg m^−^^3^
Monoclinic, *C*2/*c*	Cu *K*α radiation, λ = 1.54178 Å
*a* = 40.2728 (8) Å	Cell parameters from 9828 reflections
*b* = 6.9679 (1) Å	θ = 2.2–68.5°
*c* = 16.0119 (3) Å	µ = 0.51 mm^−^^1^
β = 92.588 (1)°	*T* = 210 K
*V* = 4488.63 (14) Å^3^	Plate, light yellow
*Z* = 8	0.25 × 0.24 × 0.05 mm

### Data collection

**Table:**

Bruker X8 Proteum diffractometer	4089 independent reflections
Radiation source: fine-focus rotating anode	3656 reflections with *I* > 2σ(*I*)
Detector resolution: 5.6 pixels mm^-1^	*R*_int_ = 0.040
φ and ω scans	θ_max_ = 68.5°, θ_min_ = 2.2°
Absorption correction: multi-scan (*SADABS*; Krause *et al.*, 2015)	*h* = −48→48
*T*_min_ = 0.822, *T*_max_ = 0.942	*k* = −8→7
28464 measured reflections	*l* = −12→19

### Refinement

**Table:**

Refinement on *F*^2^	Secondary atom site location: difference Fourier map
Least-squares matrix: full	Hydrogen site location: difference Fourier map
*R*[*F*^2^ > 2σ(*F*^2^)] = 0.038	H-atom parameters constrained
*wR*(*F*^2^) = 0.120	*w* = 1/[σ^2^(*F*_o_^2^) + (0.0642*P*)^2^ + 1.3326*P*] where *P* = (*F*_o_^2^ + 2*F*_c_^2^)/3
*S* = 1.08	(Δ/σ)_max_ = 0.001
4089 reflections	Δρ_max_ = 0.15 e Å^−^^3^
272 parameters	Δρ_min_ = −0.14 e Å^−^^3^
0 restraints	Extinction correction: *SHELXL2014* (Sheldrick, 2015), Fc^*^=kFc[1+0.001xFc^2^λ^3^/sin(2θ)]^-1/4^
Primary atom site location: structure-invariant direct methods	Extinction coefficient: 0.00034 (8)

### Special details

**Table:**

Experimental. The crystal was mounted using polyisobutene oil on the tip of a fine glass fibre, which was fastened in a copper mounting pin with electrical solder. It was placed directly into the cold gas stream of a liquid-nitrogen based cryostat (Hope, 1994; Parkin & Hope, 1998). At 90K the diffraction pattern showed some diffuse scatter and the Bragg diffraction spots were fuzzy. Visual inspection of crystal integrity and diffraction quality vs temperature established a safe temperature for data collection of -63° C.
Geometry. All esds (except the esd in the dihedral angle between two l.s. planes) are estimated using the full covariance matrix. The cell esds are taken into account individually in the estimation of esds in distances, angles and torsion angles; correlations between esds in cell parameters are only used when they are defined by crystal symmetry. An approximate (isotropic) treatment of cell esds is used for estimating esds involving l.s. planes.
Refinement. Refinement progress was checked using *Platon* (Spek, 2009) and by an *R*-tensor (Parkin, 2000). The final model was further checked with the IUCr utility *checkCIF*.

### Fractional atomic coordinates and isotropic or equivalent isotropic displacement parameters (Å^2^)

**Table:**

	*x*	*y*	*z*	*U*_iso_*/*U*_eq_	
N1	0.09256 (2)	0.51979 (13)	0.25747 (5)	0.0358 (2)	
N2	0.40754 (2)	0.51181 (13)	0.99141 (5)	0.0375 (2)	
C1	0.08446 (3)	0.34286 (16)	0.30095 (7)	0.0408 (3)	
H1A	0.0960	0.3411	0.3561	0.049*	
H1B	0.0921	0.2325	0.2692	0.049*	
C2	0.04725 (3)	0.32626 (19)	0.31130 (8)	0.0510 (3)	
H2A	0.0358	0.3157	0.2562	0.061*	
H2B	0.0426	0.2097	0.3429	0.061*	
C3	0.03424 (3)	0.4991 (2)	0.35658 (8)	0.0531 (3)	
H3A	0.0432	0.4996	0.4145	0.064*	
H3B	0.0100	0.4920	0.3576	0.064*	
C4	0.04421 (3)	0.6818 (2)	0.31330 (8)	0.0524 (3)	
H4A	0.0375	0.7929	0.3461	0.063*	
H4B	0.0327	0.6893	0.2581	0.063*	
C5	0.08159 (3)	0.68738 (16)	0.30341 (7)	0.0429 (3)	
H5A	0.0874	0.8043	0.2734	0.051*	
H5B	0.0930	0.6906	0.3587	0.051*	
C6	0.12794 (3)	0.52990 (17)	0.24185 (7)	0.0408 (3)	
H6A	0.1319	0.6458	0.2091	0.049*	
H6B	0.1337	0.4192	0.2077	0.049*	
C7	0.15040 (3)	0.53310 (16)	0.31739 (7)	0.0390 (3)	
C8	0.16846 (3)	0.53582 (15)	0.37892 (7)	0.0363 (3)	
C9	0.19232 (3)	0.53774 (13)	0.44832 (6)	0.0326 (2)	
C10	0.22585 (3)	0.50840 (14)	0.43383 (6)	0.0335 (2)	
H10A	0.2324	0.4867	0.3790	0.040*	
C11	0.24966 (2)	0.51066 (14)	0.49867 (6)	0.0313 (2)	
H11A	0.2721	0.4898	0.4873	0.038*	
C12	0.24091 (2)	0.54353 (13)	0.58071 (6)	0.0284 (2)	
C13	0.20725 (2)	0.57162 (15)	0.59484 (6)	0.0355 (2)	
H13A	0.2007	0.5932	0.6497	0.043*	
C14	0.18335 (3)	0.56848 (16)	0.53020 (6)	0.0378 (3)	
H14A	0.1609	0.5872	0.5416	0.045*	
C15	0.26622 (2)	0.54789 (13)	0.65079 (6)	0.0281 (2)	
C16	0.26055 (2)	0.64996 (14)	0.72383 (6)	0.0326 (2)	
H16A	0.2409	0.7220	0.7272	0.039*	
C17	0.28313 (2)	0.64730 (14)	0.79115 (6)	0.0331 (2)	
H17A	0.2786	0.7173	0.8395	0.040*	
C18	0.31256 (2)	0.54258 (14)	0.78869 (6)	0.0316 (2)	
C19	0.31910 (2)	0.44566 (15)	0.71481 (6)	0.0337 (2)	
H19A	0.3391	0.3779	0.7108	0.040*	
C20	0.29630 (2)	0.44858 (14)	0.64742 (6)	0.0312 (2)	
H20A	0.3012	0.3823	0.5983	0.037*	
C21	0.33448 (2)	0.53494 (15)	0.86180 (6)	0.0352 (2)	
C22	0.35066 (3)	0.53208 (16)	0.92647 (7)	0.0390 (3)	
C23	0.37192 (3)	0.52602 (18)	1.00433 (7)	0.0424 (3)	
H23A	0.3679	0.6422	1.0368	0.051*	
H23B	0.3653	0.4158	1.0377	0.051*	
C24	0.41943 (3)	0.68044 (16)	0.94747 (7)	0.0413 (3)	
H24A	0.4138	0.7965	0.9784	0.050*	
H24B	0.4083	0.6878	0.8919	0.050*	
C25	0.45676 (3)	0.67152 (19)	0.93867 (7)	0.0498 (3)	
H25A	0.4640	0.7830	0.9069	0.060*	
H25B	0.4680	0.6759	0.9942	0.060*	
C26	0.46631 (3)	0.48932 (19)	0.89439 (8)	0.0519 (3)	
H26A	0.4906	0.4800	0.8937	0.062*	
H26B	0.4575	0.4927	0.8364	0.062*	
C27	0.45268 (3)	0.3159 (2)	0.93827 (8)	0.0528 (3)	
H27A	0.4638	0.3029	0.9937	0.063*	
H27B	0.4572	0.2000	0.9061	0.063*	
C28	0.41548 (3)	0.33533 (17)	0.94746 (7)	0.0434 (3)	
H28A	0.4042	0.3357	0.8920	0.052*	
H28B	0.4074	0.2248	0.9784	0.052*	

### Atomic displacement parameters (Å^2^)

**Table:**

	*U*^11^	*U*^22^	*U*^33^	*U*^12^	*U*^13^	*U*^23^
N1	0.0341 (5)	0.0429 (5)	0.0297 (4)	−0.0006 (4)	−0.0047 (3)	0.0028 (3)
N2	0.0329 (5)	0.0501 (6)	0.0288 (4)	−0.0020 (4)	−0.0046 (3)	0.0012 (4)
C1	0.0456 (6)	0.0379 (6)	0.0388 (6)	−0.0012 (5)	0.0003 (4)	−0.0013 (4)
C2	0.0479 (7)	0.0545 (7)	0.0509 (7)	−0.0114 (6)	0.0054 (5)	−0.0028 (6)
C3	0.0438 (7)	0.0675 (8)	0.0488 (7)	0.0022 (6)	0.0096 (5)	0.0003 (6)
C4	0.0492 (7)	0.0564 (8)	0.0514 (7)	0.0146 (6)	0.0012 (5)	0.0027 (6)
C5	0.0485 (6)	0.0378 (6)	0.0418 (6)	0.0030 (5)	−0.0050 (5)	0.0029 (5)
C6	0.0363 (6)	0.0528 (7)	0.0326 (5)	−0.0018 (5)	−0.0045 (4)	0.0057 (5)
C7	0.0356 (6)	0.0426 (6)	0.0382 (6)	−0.0008 (4)	−0.0042 (5)	0.0025 (4)
C8	0.0361 (6)	0.0345 (6)	0.0379 (6)	0.0006 (4)	−0.0034 (4)	0.0011 (4)
C9	0.0348 (5)	0.0269 (5)	0.0356 (5)	−0.0001 (4)	−0.0053 (4)	0.0016 (4)
C10	0.0388 (5)	0.0324 (5)	0.0293 (5)	−0.0012 (4)	0.0001 (4)	−0.0003 (4)
C11	0.0299 (5)	0.0302 (5)	0.0338 (5)	−0.0006 (4)	0.0016 (4)	−0.0001 (4)
C12	0.0312 (5)	0.0221 (5)	0.0315 (5)	0.0001 (3)	−0.0002 (4)	0.0012 (4)
C13	0.0335 (5)	0.0411 (6)	0.0319 (5)	0.0060 (4)	0.0009 (4)	−0.0022 (4)
C14	0.0304 (5)	0.0434 (6)	0.0393 (6)	0.0064 (4)	−0.0013 (4)	−0.0007 (5)
C15	0.0293 (5)	0.0249 (5)	0.0300 (5)	−0.0009 (4)	0.0008 (4)	0.0020 (4)
C16	0.0319 (5)	0.0316 (5)	0.0343 (5)	0.0047 (4)	0.0014 (4)	−0.0016 (4)
C17	0.0351 (5)	0.0330 (5)	0.0310 (5)	−0.0002 (4)	0.0005 (4)	−0.0033 (4)
C18	0.0298 (5)	0.0300 (5)	0.0346 (5)	−0.0044 (4)	−0.0020 (4)	0.0020 (4)
C19	0.0276 (5)	0.0342 (5)	0.0391 (5)	0.0032 (4)	−0.0006 (4)	−0.0011 (4)
C20	0.0304 (5)	0.0309 (5)	0.0323 (5)	0.0014 (4)	0.0017 (4)	−0.0033 (4)
C21	0.0312 (5)	0.0365 (6)	0.0375 (6)	−0.0030 (4)	−0.0015 (4)	0.0005 (4)
C22	0.0330 (5)	0.0461 (6)	0.0374 (6)	−0.0026 (4)	−0.0026 (4)	−0.0007 (4)
C23	0.0344 (6)	0.0607 (7)	0.0317 (5)	−0.0029 (5)	−0.0031 (4)	−0.0023 (5)
C24	0.0420 (6)	0.0419 (6)	0.0394 (6)	−0.0032 (5)	−0.0036 (4)	−0.0013 (5)
C25	0.0415 (6)	0.0599 (8)	0.0479 (7)	−0.0104 (5)	−0.0007 (5)	0.0020 (5)
C26	0.0401 (7)	0.0682 (8)	0.0480 (7)	0.0049 (6)	0.0079 (5)	0.0065 (6)
C27	0.0494 (7)	0.0566 (8)	0.0523 (7)	0.0125 (6)	0.0025 (5)	0.0084 (6)
C28	0.0464 (6)	0.0425 (6)	0.0408 (6)	0.0004 (5)	−0.0026 (5)	0.0045 (5)

### Geometric parameters (Å, º)

**Table:**

N1—C5	1.4592 (14)	C13—C14	1.3814 (14)
N1—C6	1.4595 (14)	C13—H13A	0.9400
N1—C1	1.4599 (14)	C14—H14A	0.9400
N2—C28	1.4596 (15)	C15—C16	1.3964 (13)
N2—C24	1.4614 (14)	C15—C20	1.3985 (13)
N2—C23	1.4616 (14)	C16—C17	1.3787 (13)
C1—C2	1.5194 (16)	C16—H16A	0.9400
C1—H1A	0.9800	C17—C18	1.3938 (14)
C1—H1B	0.9800	C17—H17A	0.9400
C2—C3	1.5113 (18)	C18—C19	1.3974 (14)
C2—H2A	0.9800	C18—C21	1.4352 (13)
C2—H2B	0.9800	C19—C20	1.3849 (13)
C3—C4	1.5126 (19)	C19—H19A	0.9400
C3—H3A	0.9800	C20—H20A	0.9400
C3—H3B	0.9800	C21—C22	1.1984 (15)
C4—C5	1.5210 (16)	C22—C23	1.4807 (14)
C4—H4A	0.9800	C23—H23A	0.9800
C4—H4B	0.9800	C23—H23B	0.9800
C5—H5A	0.9800	C24—C25	1.5176 (15)
C5—H5B	0.9800	C24—H24A	0.9800
C6—C7	1.4776 (14)	C24—H24B	0.9800
C6—H6A	0.9800	C25—C26	1.5123 (18)
C6—H6B	0.9800	C25—H25A	0.9800
C7—C8	1.1980 (15)	C25—H25B	0.9800
C8—C9	1.4360 (13)	C26—C27	1.5133 (18)
C9—C14	1.3921 (15)	C26—H26A	0.9800
C9—C10	1.3956 (14)	C26—H26B	0.9800
C10—C11	1.3810 (14)	C27—C28	1.5177 (16)
C10—H10A	0.9400	C27—H27A	0.9800
C11—C12	1.3944 (14)	C27—H27B	0.9800
C11—H11A	0.9400	C28—H28A	0.9800
C12—C13	1.3979 (13)	C28—H28B	0.9800
C12—C15	1.4817 (13)		
			
C5—N1—C6	111.59 (9)	C13—C14—C9	120.42 (9)
C5—N1—C1	110.87 (8)	C13—C14—H14A	119.8
C6—N1—C1	111.31 (8)	C9—C14—H14A	119.8
C28—N2—C24	111.19 (8)	C16—C15—C20	117.33 (9)
C28—N2—C23	111.30 (9)	C16—C15—C12	120.78 (8)
C24—N2—C23	111.03 (9)	C20—C15—C12	121.89 (8)
N1—C1—C2	111.04 (9)	C17—C16—C15	121.39 (9)
N1—C1—H1A	109.4	C17—C16—H16A	119.3
C2—C1—H1A	109.4	C15—C16—H16A	119.3
N1—C1—H1B	109.4	C16—C17—C18	121.11 (9)
C2—C1—H1B	109.4	C16—C17—H17A	119.4
H1A—C1—H1B	108.0	C18—C17—H17A	119.4
C3—C2—C1	110.89 (10)	C17—C18—C19	118.03 (9)
C3—C2—H2A	109.5	C17—C18—C21	119.27 (9)
C1—C2—H2A	109.5	C19—C18—C21	122.69 (9)
C3—C2—H2B	109.5	C20—C19—C18	120.59 (9)
C1—C2—H2B	109.5	C20—C19—H19A	119.7
H2A—C2—H2B	108.0	C18—C19—H19A	119.7
C2—C3—C4	110.24 (10)	C19—C20—C15	121.48 (9)
C2—C3—H3A	109.6	C19—C20—H20A	119.3
C4—C3—H3A	109.6	C15—C20—H20A	119.3
C2—C3—H3B	109.6	C22—C21—C18	174.81 (11)
C4—C3—H3B	109.6	C21—C22—C23	177.50 (11)
H3A—C3—H3B	108.1	N2—C23—C22	114.63 (9)
C3—C4—C5	110.71 (10)	N2—C23—H23A	108.6
C3—C4—H4A	109.5	C22—C23—H23A	108.6
C5—C4—H4A	109.5	N2—C23—H23B	108.6
C3—C4—H4B	109.5	C22—C23—H23B	108.6
C5—C4—H4B	109.5	H23A—C23—H23B	107.6
H4A—C4—H4B	108.1	N2—C24—C25	111.07 (9)
N1—C5—C4	110.83 (9)	N2—C24—H24A	109.4
N1—C5—H5A	109.5	C25—C24—H24A	109.4
C4—C5—H5A	109.5	N2—C24—H24B	109.4
N1—C5—H5B	109.5	C25—C24—H24B	109.4
C4—C5—H5B	109.5	H24A—C24—H24B	108.0
H5A—C5—H5B	108.1	C26—C25—C24	110.61 (10)
N1—C6—C7	115.27 (9)	C26—C25—H25A	109.5
N1—C6—H6A	108.5	C24—C25—H25A	109.5
C7—C6—H6A	108.5	C26—C25—H25B	109.5
N1—C6—H6B	108.5	C24—C25—H25B	109.5
C7—C6—H6B	108.5	H25A—C25—H25B	108.1
H6A—C6—H6B	107.5	C25—C26—C27	110.31 (10)
C8—C7—C6	179.62 (12)	C25—C26—H26A	109.6
C7—C8—C9	175.36 (11)	C27—C26—H26A	109.6
C14—C9—C10	118.25 (9)	C25—C26—H26B	109.6
C14—C9—C8	122.49 (9)	C27—C26—H26B	109.6
C10—C9—C8	119.26 (9)	H26A—C26—H26B	108.1
C11—C10—C9	121.15 (9)	C26—C27—C28	110.76 (10)
C11—C10—H10A	119.4	C26—C27—H27A	109.5
C9—C10—H10A	119.4	C28—C27—H27A	109.5
C10—C11—C12	120.92 (9)	C26—C27—H27B	109.5
C10—C11—H11A	119.5	C28—C27—H27B	109.5
C12—C11—H11A	119.5	H27A—C27—H27B	108.1
C11—C12—C13	117.61 (9)	N2—C28—C27	111.13 (9)
C11—C12—C15	121.50 (8)	N2—C28—H28A	109.4
C13—C12—C15	120.90 (8)	C27—C28—H28A	109.4
C14—C13—C12	121.63 (9)	N2—C28—H28B	109.4
C14—C13—H13A	119.2	C27—C28—H28B	109.4
C12—C13—H13A	119.2	H28A—C28—H28B	108.0
			
C5—N1—C1—C2	59.37 (11)	C11—C12—C15—C20	−26.18 (13)
C6—N1—C1—C2	−175.79 (9)	C13—C12—C15—C20	153.55 (10)
N1—C1—C2—C3	−56.43 (13)	C20—C15—C16—C17	−2.33 (14)
C1—C2—C3—C4	53.55 (14)	C12—C15—C16—C17	176.66 (8)
C2—C3—C4—C5	−53.83 (14)	C15—C16—C17—C18	0.11 (15)
C6—N1—C5—C4	175.67 (9)	C16—C17—C18—C19	2.24 (14)
C1—N1—C5—C4	−59.65 (11)	C16—C17—C18—C21	−176.50 (9)
C3—C4—C5—N1	57.03 (13)	C17—C18—C19—C20	−2.32 (14)
C5—N1—C6—C7	62.09 (12)	C21—C18—C19—C20	176.37 (9)
C1—N1—C6—C7	−62.35 (12)	C18—C19—C20—C15	0.08 (15)
C14—C9—C10—C11	−0.34 (14)	C16—C15—C20—C19	2.24 (14)
C8—C9—C10—C11	179.46 (9)	C12—C15—C20—C19	−176.74 (8)
C9—C10—C11—C12	−0.38 (14)	C28—N2—C23—C22	−61.65 (12)
C10—C11—C12—C13	0.76 (14)	C24—N2—C23—C22	62.79 (12)
C10—C11—C12—C15	−179.51 (8)	C28—N2—C24—C25	−58.93 (11)
C11—C12—C13—C14	−0.43 (15)	C23—N2—C24—C25	176.57 (9)
C15—C12—C13—C14	179.83 (9)	N2—C24—C25—C26	56.67 (12)
C12—C13—C14—C9	−0.29 (16)	C24—C25—C26—C27	−54.12 (13)
C10—C9—C14—C13	0.67 (15)	C25—C26—C27—C28	53.94 (14)
C8—C9—C14—C13	−179.13 (9)	C24—N2—C28—C27	58.70 (11)
C11—C12—C15—C16	154.88 (10)	C23—N2—C28—C27	−176.96 (9)
C13—C12—C15—C16	−25.40 (13)	C26—C27—C28—N2	−56.26 (13)
